# miR-129-5p restores cardiac function in rats with chronic heart failure by targeting the E3 ubiquitin ligase Smurf1 and promoting PTEN expression

**DOI:** 10.1080/21655979.2021.2024335

**Published:** 2022-01-16

**Authors:** Yuan Qi, Yan Tang, Lu Yin, Keke Ding, Cuimei Zhao, Wenwen Yan, Yi’an Yao

**Affiliations:** a Department of Cardiology, Gongli Hospital of Shanghai Pudong New Area, Shanghai, China; b Department of Cardiology, Tongji Hospital Affiliated to Tongji University, Shanghai, China

**Keywords:** Chronic heart failure, miR-129-5p, E3 ubiquitin ligase Smurf1, PTEN, ubiquitination, cardiac dysfunction

## Abstract

Chronic heart failure (CHF) is a prevalent health concern with complex pathogenesis. This current study set out to estimate the function of the miR-129-5p/Smurf1/PTEN axis on cardiac function injury in CHF. The model of CHF in rats was established. The cardiac function indexes, myocardial tissue damage, and oxidative stress-related factors in CHF rats were evaluated after the interference of Smurf1/miR-129-5p/PTEN. The targeting relationships between miR-129-5p and Smurf1 and between PTEN and Smurf1 were verified. It was found that that after modeling, cardiac functions were impaired, heart/left ventricular/lung weight and the myocardial structure was destroyed, and the degree of fibrosis of myocardial tissue was increased. After Smurf1 knockdown, the cardiac function, myocardial structure, and oxidative stress were improved, and the fibrosis in myocardial tissue was decreased. Smurf1 was a target of miR-129-5p. miR-129-5p could annul the protective effect of Smurf1 silencing on CHF rats. Smurf1 inhibited PTEN expression by promoting PTEN ubiquitination, while miR-129-5p enhanced PTEN expression by inhibiting Smurf1. Meanwhile, overexpression of PTEN annulled the cardiac dysfunction in CHF rats induced by Smurf1. In conclusion, miR-129-5p targeted Smurf1 and repressed the ubiquitination of PTEN, and promoted PTEN expression, thus improving the cardiac function of CHF rats.

## Graphical abstract

**Figure:**
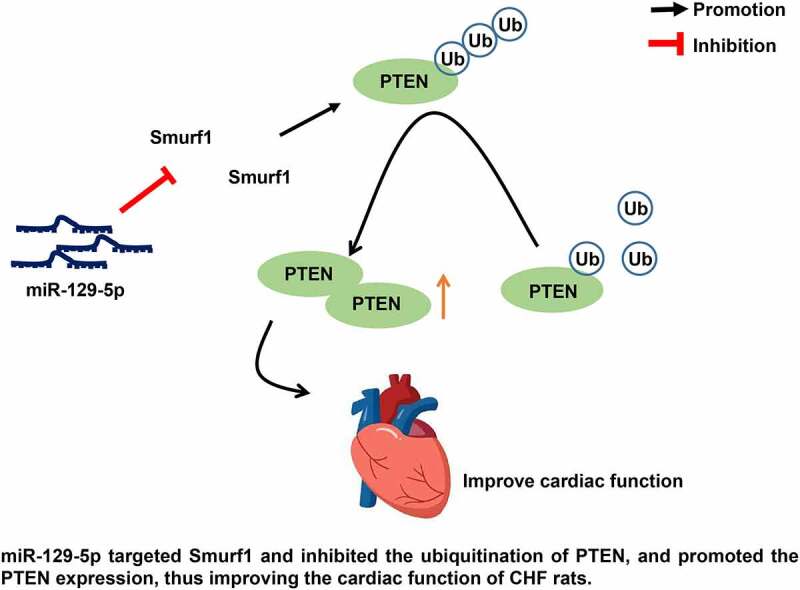

## 1. Introduction

Chronic heart failure (CHF) is a progressive clinical condition and a life-threatening major challenge that is closely associated with increased morbidity and mortality worldwide [1]. In recent decades, despite great improvements in the management of heart failure in terms of medical intervention and equipment, the incidence of CHF is still going up, and the quality of life, cardiac function, and life expectancy of affected patients decline to different degrees of impairment [2]. Typical symptoms of CHF include dyspnea, limb swelling, and fatigue [3]. The hard-done work of our peers has shown the association between CHF and myocardial and oxidative stress [4]. CHF has become a well-known public health problem, but its pathogenesis and progression are very complex and varied [5]. Therefore, it is imperative to further elaborate the underlying pathogenesis and progression of CHF and develop novel efficacious strategies to intervene CHF.

Human Smurf1 (Smad ubiquitin regulatory factor 1) is a tumor promoter by ubiquitination modification and/or degradation of tumor-suppressing proteins, and it is principally expressed in the nervous system, bone, lung, reproductive organs, and other tissues [6]. More importantly, recent data suggest that Smurf1 is associated with cardiovascular diseases [7]. Raised Activin type II receptor signaling links aging and the pathobiology of heart failure through the regulation of follistatin-like 3 and Smurf1 [7]. But the expression pattern and role of Smurf1 in CHF is largely elusive.

On a separate note, Smurf1 ubiquitylates and degrades phosphatase and tensin homolog (PTEN) in glioblastoma [8]. Down-regulation of PTEN activates the Akt/mTOR and TGF-βRI/Smad2 signaling pathways in human cardiac fibroblasts, accelerating proliferation of human cardiac fibroblasts and myocardial fibrosis, thereby leading to CHF [9]. PTEN could attenuate myocardial hypertrophy and cardiac dysfunction mediated by miR-217 overexpression in the heart of CHF patients [10]. Nevertheless, whether Smurf1 modulates PTEN ubiquitination in CHF rats is unidentified.

microRNAs (miRNAs) are small endogenous non-coding RNAs that are essential tools for regulating gene expression and are involved in a variety of biological events, like cell proliferation, differentiation, and apoptosis [11]. Smurf1 is reported to be a downstream target of miRNAs and is implicated in miRNAs-mediated disease progression [12]. Circulating miRNAs are noted as diagnostic and prognostic biomarkers for several diseases, including heart failure [13]. There is evidence to suggest that miR-129-5p can improve cardiac function in CHF rats and inhibit the apoptosis of cardiomyocytes [14]. miR-129-5p ameliorates cardiac function in rats with CHF by targeting HMGB1 [15]. The suppressed expression of miR-129-5p was found in the angiotensin II–induced cardiomyocyte hypertrophy, a main cause of heart failure [16]. miR-129-5p expression is reduced in CHF patients, and it can be used as a noninvasive diagnostic and prognostic biomarker for CHF [14]. However, the mechanism of miR-129-5p regulating Smurf1 in CHF has not been reported at home and abroad. Therefore, our strategy was to explore the regulatory effect of miR-129-5p/Smurf1 on cardiac function damage in CHF. By establishing a rat model of CHF, we found that miR-129-5p inhibited the ubiquitination of PTEN by targeting Smurf1, thereby promoting the expression of PTEN and improving the cardiac function of CHF.

## 2. Materials and methods

### 2.1. Ethics statements

Animal experiments conformed to the standards established by the animal experiment committee of Tongji Hospital affiliated to Tongji University and were approved by the ethics committee of Tongji Hospital affiliated to Tongji University.

### 2.2. Establishment of rat models of CHF

Male Wistar rats (N = 130, 6 weeks, 200–300 g) from Hunan SJA Laboratory Animal Co. Ltd (Hunan, China) were placed in 12-h light/dark cycles with *ad libitum* access to food and water. The CHF model was established by ligation of the anterior descending branch of the left coronary artery. The control rats were treated with the same surgical method without arterial ligation. Four weeks after arterial ligation, the rats were allocated to 13 groups with 10 rats per group as follows: control group, CHF group (model establishment), sh-NC group (after modeling, rats were injected with the negative control of Smurf1 lentivirus interference vectors), sh-Smurf1 group (after modeling, rats were injected with Smurf1 lentivirus interference vectors), agomiR-NC+oe-NC group (rats were injected with of miR-129-5p agomiR-NC and negative control of Smurf1 overexpression vectors), agomiR-NC + oe-Smurf1 group (after modeling, rats were injected with miR-129-5p agomiR-NC and Smurf1 overexpression vectors), miR-129-5p agomiR + oe NC group (after modeling, rats were injected with miR-129-5p agomiR and negative control of Smurf1 overexpression vectors), miR-129-5p agomiR + oe-Smurf1 group (rats were injected with miR-129-5p agomiR and Smurf1 overexpression vectors), oe-NC group (after modeling, rats were injected with negative control of Smurf1 overexpression vector and negative control of PTEN overexpression vector), oe-NC + oe-PTEN group (after modeling, rats were injected with negative control of Smurf1 overexpression vector and PTEN overexpression vector), oe-Smurf1+ oe-NC group (after modeling, rats were injected with Smurf1 overexpression vectors and negative control of PTEN overexpression vectors), and oe-Smurf1 + oe-PTEN group (after modeling, rats were injected with Smurf1 and PTEN overexpression vectors). Four weeks after injection, transthoracic echocardiography was performed to assess the heart function. After that, the rats were anesthetized using 50 mg/kg pentobarbital sodium to remove the heart and lung tissues.

### 2.3. Heart function detection

The Vevo 2100 system (FUJIFILM VisualSonics Inc., Toronto, Canada) was used to display the left ventricular end-diastolic dimension (LVEDD), left ventricular end-systolic dimension (LVESD), ejection fraction (EF), and fractional shortening (FS). The calculation formula is as follows: EF (%) =  [(EDD^3^  −  ESD^3^)/EDD^3^]  ×  100; FS (%) =  [(LVEDD − LVESD)/LVEDD]  ×  100. All values were continuously measured for 6 consecutive cardiac cycles in the same rat. The rats were selected blindly.

### 2.4. Hematoxylin-eosin (HE) staining

The tissue sections were flattened and pasted on the slides, dried, and dewaxed, then dehydrated with alcohol from high concentration to low concentration, and washed in distilled water for 5 minutes. Next, the sections were stained with hematoxylin (517–28-2, Solarbio, Beijing, China) for 5 minutes, differentiated with 1% hydrochloric acid and ethanol for 3 seconds, and stained with 5% eosin solution (17,372–87-1, Solarbio). After that, the sections were dehydrated, cleared, sealed, and observed [17].

### 2.5. Masson staining

After dewaxing, sections were sliced, stained with ponceau solution for 2 minutes, immersed in 0.2% glacial acetic acid solution, differentiated in 5% phosphomolybdate solution, and immersed in 0.2% glacial acetic acid solution, and stained with methyl green dye. Finally, the sections were sealed with neutral gum and observed [18].

### 2.6. Immunohistochemical staining

Paraffined specimens of each group were collected, sliced at a thickness of 4 μm), dewaxed, and steps were operated according to the conventional immunohistochemical staining method. The primary rabbit anti-Smurf1 (ab57573; 1:1000; Abcam; UK) was added for incubation. Staining results were determined by randomly selecting 5 lesion areas at high magnification and counting the number of cells with positive staining. Results can be calculated according to the percentage of positive cells. Each experiment was conducted 3 times [19–21].

### 2.7. Oxidative stress detection

The myocardial tissue of the left ventricular was minced evenly, and 1 g tissue was resuspended in 2 mL RIPA lysis buffer. Then, the activity of superoxide dismutase (SOD), malondialdehyde (MDA), and glutathione peroxidase (GSH-Px) was measured using enzyme-linked immunosorbent assay (ELISA) kits (Solarbio). Finally, the absorbance was measured using a microplate reader (Millipore, Temecula, CA, USA) [22].

### 2.8. Dual-luciferase reporter assay

Synthetized Smurf1 3ʹUTR fragments were inserted into the pGL3-reporter (Promega, WI, USA) using endonuclease sites XhoI and BamH I to design complementary sequence mutation sites on Smurf1 wild-type seed sequences. The target fragment was inserted into the pGL3-reporter using a T4 DNA ligase after digestion with restriction enzymes. The constructed Smurf1 wild-type (wt) or mutant type (mut) was co-transfected into HEK293T cells with miR-129-5p mimic or mimic-NC. After 48 hours, the cells were lysed and luciferase activity was testified using the dual-luciferase assay kit (Promega) on the Luminometer TD-20/20 detector (Promega Biotech), respectively [17].

### 2.9. Cell culture and transfection

Primary rat cardiomyocytes were provided by ATCC (Shanghai, China). To produce cells overexpressing the Smurf1 (the Smurf1 group), cells were delivered with the Smurf1 plasmid for 8 hours using 5 µL Lipofectamine 3000 (Invitrogen Co., LA, USA). Next, cells were cotransfected with Smurf1 plasmids and miR-129-5p mimic (30 µmoL; Riobio, Guangzhou, China) to produce the Smurf1 + miR-129-5p group. The proteasome inhibitor MG132 (10 µM) or DMSO (0.1%) was used as solvent control. They were added to the cell culture medium 2 hours prior to mechanical strain application and remained in the culture medium throughout the experiment [23].

### 2.10. Quantitative reverse transcription polymerase chain reaction (qRT-PCR)

Cardiomyocytes were collected and the total RNA content was extracted using the TRIzol reagent (Invitrogen). The first-strand cDNA synthesis kit (Takara Bio INC., Otsu, Japan) was used to synthesize the first strand of cDNA by reverse transcription. The gene expression was tested by qRT-PCR using the SYBR Premix Ex Taq kit (Takara Bio). The PCR was performed using ABI Prism 7500 Fast Real-Time PCR System (Applied Biosystems, MA, USA) under the subsequent conditions: pre-denaturation at 95°C for 10 minutes, and 40 cycles of denaturation at 94°C for 30 seconds, annealing at 59°C for 30 seconds and extension at 72°C for 30 seconds. Gene expression was calculated based on the 2^−ΔΔCt^ method. The internal parameters were U6 and GAPDH, and the primer sequences are shown in Table 1. When detecting the RNA in rat myocardial tissue samples, 0.1 g tissue samples were weighed and ground at 4°C with a tissue grinding machine (KZ-II, Serice Biotechnology Co. Ltd., Wuhan, China). After that, RNA was extracted using TRIzol. The procedure was the same as above [17].

**Table 1.** Primer sequences for qRT-PCR

Gene	Primer sequences
miR-129-5p	F: 5ʹ-GATCCGCAAGCCCAGACCGCAAAAAGTTTTTA-3’
	R: 5ʹ-AGCTTAAAAACTTTTTGCGGTCTGGGCTTGCG-3’
Smurf1	F: 5ʹ-AGTTCGTGGCCAAATAGTGG-3’
	R: 5ʹ-GTTCCTTCGTTCTCCAGCAG-3’
PTEN	F: 5ʹ-CAGATTATGGAATGTAGGCGGCTTGA-3’
	R: 5ʹ-TGGCAATAGCCGAACAGTTCT-3’
GAPDH	F: 5ʹ-AGTGCCAGCCTCGTCTCATA-3’
	R: 5ʹ-GGTAACCAGGCGTCCGATAC-3’
U6	F: 5ʹ-CTCGCTTCGGCAGCACA-3’
	R: 5ʹ-AACGCTTCACGAATTTGCGT-3’

### 2.11. Western blot (WB)

The total protein was extracted using RIPA (R0010, SolarBio). An appropriate amount of protein lysate was supplemented, collected in an EP tube, and placed on ice for lysis for 30 minutes. The supernatant was collected and protein concentration was determined using the bicinchoninic acid (BCA) kit (P0011, Beyotime Biotechnology, Shanghai, China). Following protein separation using polyacrylamide gel electrophoresis (PAGE), the protein transferred onto 0.2 μm polyvinylidene fluoride (PVDF) membranes (ISEQ10100, Meliore, MA, USA). The antibody was diluted with tris-buffered saline-tween buffer containing 1% skim milk powder, which was used as the primary antibody diluent and added to the membranes and incubated overnight at 4°C. The next day, the horseradish peroxidase-labeled goat anti-rabbit IgG antibody (1:5000, A0208, Beyotime) was diluted with TBST containing 1% skim milk powder, which was used as the secondary antibody diluent. The PVDF membranes were incubated at room temperature for 1 hour and detected using a digital chemiluminescence analyzer (LI-COR Bioscience, NE, USA) and analyzed using Image J. GAPDH was used as an internal reference, and the ratio of the gray value of the target band to that of GAPDH band was used as the relative level of proteins. The primary antibodies used, including rabbit anti-Smurf1 (ab236081; 1:1000), PTEN (ab267787; 1:1000), and GAPDH (ab8245; 1:1000), were all from Abcam (Cambridge, MA, USA) [24].

### 2.12. Immunocoprecipitation (IP) experiment

Cells in each group were lysed in a lysate buffer (a mixture of 50 mM Tris-HCl (pH 7.4), 150 mM NaCl, 10% glycerin, 1 mM ethylene diamine tetraacetic acid (EDTA), 0.5% NP-40, and protease inhibitors), and cell debris was removed following centrifugation. After the measurement of lysate concentration using BCA kits, the same amount of proteins was taken from each group and supplemented with cell lysate to the same volume, then 1 μg anti-Smurf1 (ab236081; 1:1000), PTEN (ab267787; 1:1000), and 15 μL protein A/G beads (Santa Cruz, Texas, USA) were added and incubated for 2 hours. After washing with cell lysate 3 times, beads were collected by centrifugation, and then an equal volume reductive loading buffer was added. The beads were boiled at 100°C for 5 minutes. Samples were separated by sodium dodecyl sulfate-PAGE, and the proteins were transferred onto PVDF membranes (Millipore), which were then analyzed by immunoblotting [25].

### 2.13. Statistical analysis

All data were processed by SPSS 21.0 (IBM Corp. Armonk, NY, USA) and tested for normal distribution and homogeneity of variance. Measurement data complied with the assumption of normal distribution and homogeneity of variance and were displayed as mean ± standard deviation. An unpaired *t*-test was used for comparisons between 2 groups. One-way ANOVA was used for comparisons among multiple groups, followed by Tukey’s test. A value of P < 0.05 was regarded statistically significant.

## 3. Results

This study was designed to investigate the effect of miR-129-5p on cardiac function of CHF rats by regulating the expression of Smurf1/PTEN. We proved that miR-129-5p may block the ubiquitination of PTEN by targeting Smurf1, thus improving the cardiac function of CHF.

### 3.1. Smurf1 was highly expressed in rats with CHF

**Figure 1.**
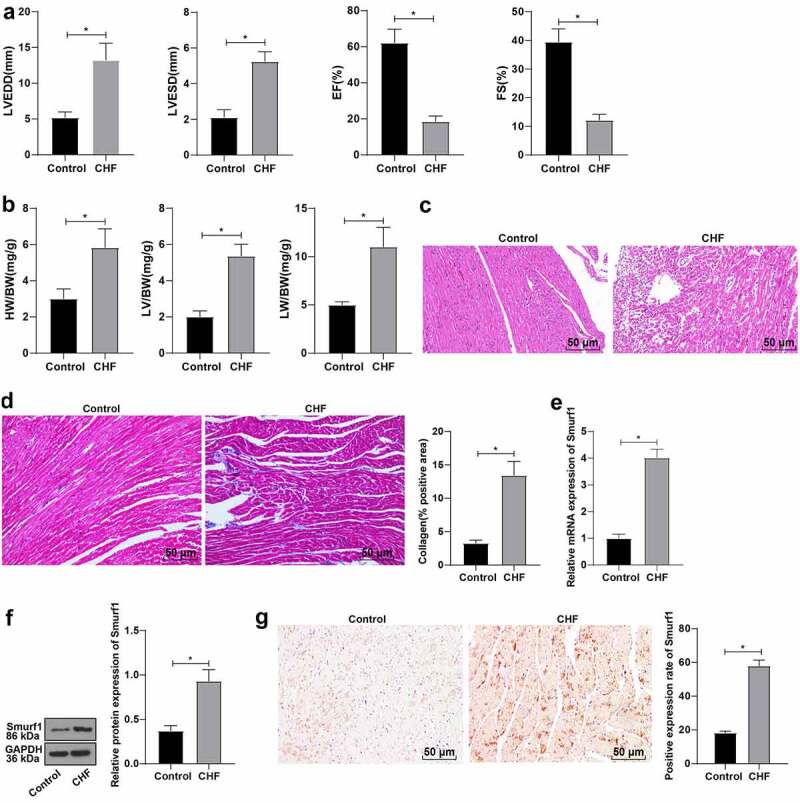
Smurf1 is highly expressed in CHF rats. A: Cardiac function indexes after modeling were detected by echocardiography; B: The ratios of heart weight/body weight, left ventricle weight/body weight, and lung weight/body weight were measured; C: Histopathological changes of the myocardium observed by HE staining; D: Masson staining observed the degree of myocardial tissue fibrosis; E: Expression of Smurf1 in myocardial tissue detected by qRT-PCR; F: Smurf1 protein level in myocardial tissue detected by WB; G: Expression of Smurf1 in the myocardium was detected by immunohistochemical staining; N = 10; Values in the figure were measurement data, which were displayed as mean ± standard deviation. Unpaired *t* test was used for comparisons. * *P* < 0.05.

Previous studies have shown that Smurf1 is overexpressed in tumors [26] and overexpression of Smurf1 can inhibit fracture healing [25,27], but there is no relevant report about the effect of Smurf1 on cardiac dysfunction in CHF rats. Firstly, we established a rat model of CHF and evaluated cardiac function indicators by echocardiography after modeling. Compared with the control group, the LVEDD and LVESD of the CHF group were raised, and the EF and FS were lowered (*P* < 0.05) (Figure 1(a)). The ratio of left ventricular weight/body weight reflects the degree of left ventricular hypertrophy, and the ratio of lung weight/body weight reflects the pulmonary edema [22]. Compared with the control group, the heart weight, left ventricular weight, and lung weight were enhanced in the CHF group (Figure 1(b)). HE staining showed that compared with the control group, the cross-sectional area of cardiomyocytes in the CHF group was enlarged and the myocardial structure was destroyed (Figure 1(c)). Masson staining showed that the proportion of fibrosis area (blue area) to myocardial surface area (red area) was markedly increased in the CHF group, and the fibrosis area was elevated obviously, indicating that the fibrosis degree of myocardial tissue in the CHF group was higher than that in the control group (Figure 1(d)). Smurf1 in the myocardium was detected by qRT-PCR and WB, which showed significantly increased Smurf1 expression levels in the myocardium of the CHF group compared with that in the control group (*P* < 0.05) (Figure 1(e,f)). Meanwhile, the immunohistochemical staining results were consistent with those of the WB experiment (Figure 1(g)). Briefly, the above-mentioned findings indicated that Smurf1 was highly expressed in CHF rats.

### 3.2. Smurf1 silencing significantly improved cardiac function in CHF rats

**Figure 2.**
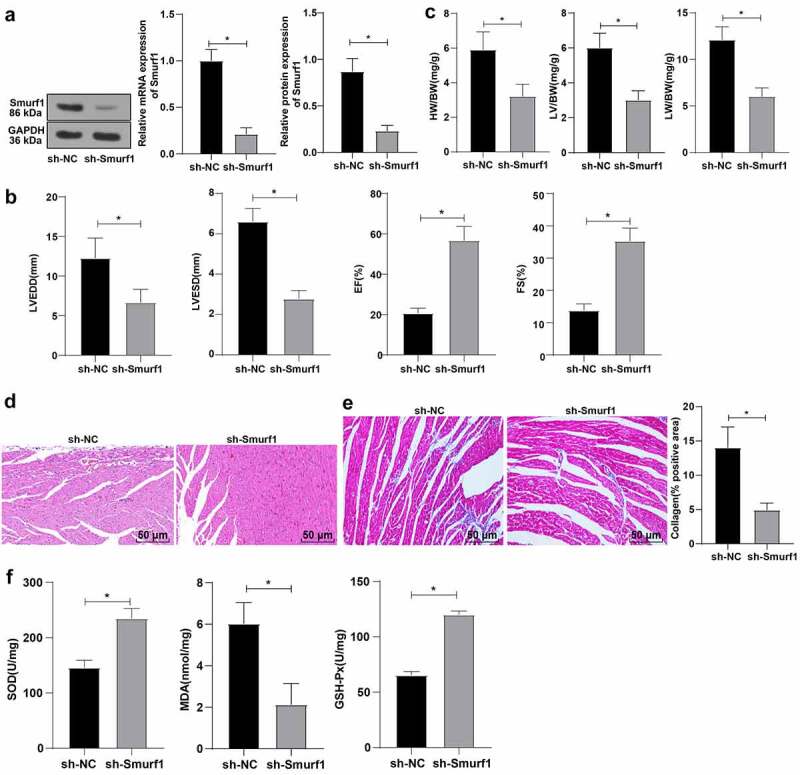
Smurf1 silencing inhibits oxidative stress levels and improves cardiac function in CHF rats. A: Smurf1 expression in myocardial tissue of rats detected by qRT-PCR and WB; B: Cardiac function indexes after modeling were detected by echocardiography; C: The ratios of heart weight/body weight, left ventricle weight/body weight, and lung weight/body weight were measured; D: Histopathological changes of the myocardium observed by HE staining; E: Masson staining observed the degree of myocardial tissue fibrosis; F: The levels of SOD, GSH-Px and MDA detected by ELISA; N = 10; Values in the figure were measurement data, which were displayed as mean ± standard deviation. Unpaired *t* test was used for comparisons. * *P* < 0.05.

Based on the above results, we concluded that Smurf1 was highly expressed in the myocardial tissues of CHF rats. Therefore, the rats after modeling were treated with Smurf1 interference, and the expression of Smurf1 in the myocardium of rats was then detected by qRT-PCR and WB, which showed that Smurf1 in the sh-Smurf1 group was lower than that in the sh-NC group (*P* < 0.05) (Figure 2(a)), indicating that Smurf1 expression was knocked down. Echocardiography showed that compared with the sh-NC group, the rats in the sh-Smurf1 group had lower LVEDD and LVESD and higher EF and FS (*P* < 0.05) (Figure 2(b)). Compared with the sh-NC group, the heart weight, left ventricular weight, and lung weight were decreased in the sh-Smurf1 group (*P* < 0.05) (Figure 2(c)). HE staining indicated that the cross-sectional area of cardiomyocytes was reduced and the myocardial structure was improved after Smurf1 knockdown (Figure 2(d)). Masson staining showed that compared with the sh-NC group, the fibrosis area (blue area) accounted for the myocardial surface area (red area) in the sh-Smurf1 group was reduced, the fibrosis area was lowered, and the degree of fibrosis in the myocardial tissue of rats was significantly decreased (Figure 2(e)). Oxidative stress levels are important in the pathophysiological process of CHF, and SOD, GSH-Px, and MDA can be used as important indexes to evaluate oxidative stress ability [22]. Therefore, we used ELISA to detect the factors related to oxidative stress. Compared with the sh-NC group, SOD and GSH-Px in the sh-Smurf1 group were raised, while MDA was decreased (*P* < 0.05) (figure 2(f)). Altogether, these findings indicated that Smurf1 silencing can inhibit oxidative stress levels and improve cardiac function in CHF rats.

### 3.3. miR-129-5p targets Smurf1

**Figure 3.**
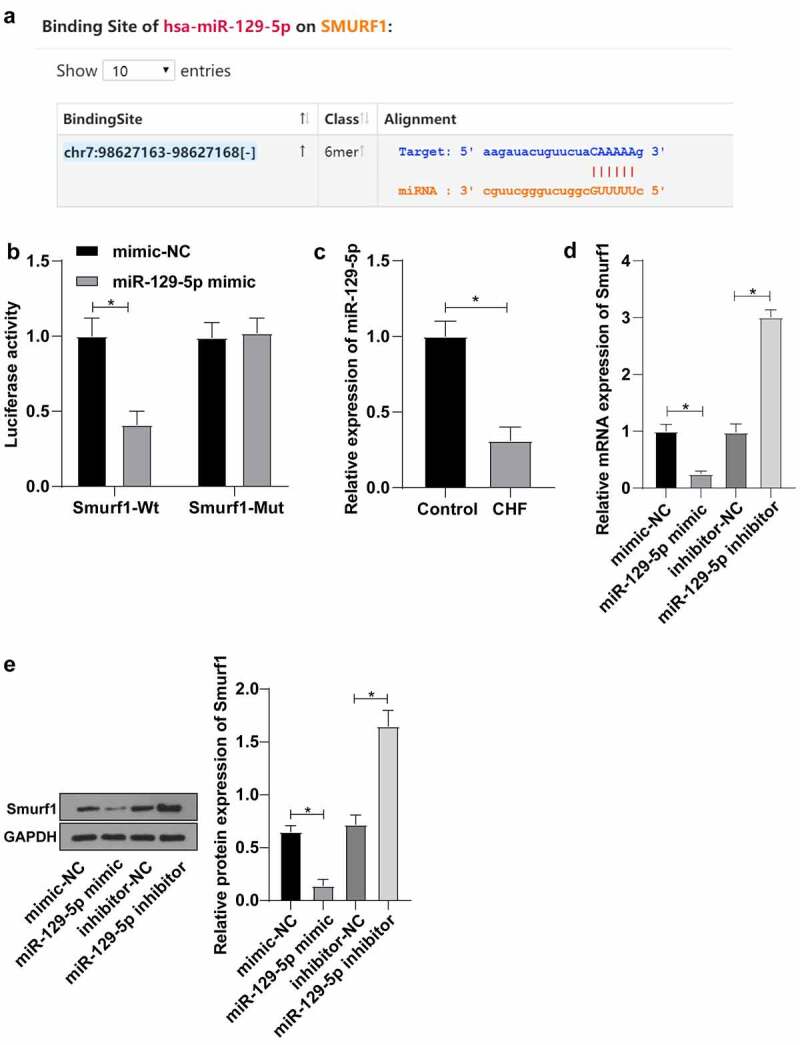
Smurf1 is the target gene of miR-129-5p. A: Starbase website predicted the targeted binding sites of miR-129-5p and Smurf1; B: The targeting relationship between miR-129-5p and Smurf1 verified by dual-luciferase assay; C: miR-129-5p expression in the myocardium of CHF rats detected by qRT-PCR; D: Smurf1 expression after miR-129-5p overexpression and interference with miR-129-5p detected by qRT-PCR; E: Smurf1 protein level detected by WB after miR-129-5p overexpression and interference with miR-129-5p; Values in the figure were measurement data and displayed as mean ± standard deviation. Unpaired *t* test was used for pairwise comparisons; one-way ANOVA was used for comparisons among multiple groups, followed by Tukey’s test. * *P* < 0.05.

To further identify the upstream mechanism of Smurf1, we predicted targeted binding sites between miR-129-5p and Smurf1 through the Starbase website (http://www.sysu.edu.cn/403.html) (Figure 3(a)). At the same time, according to the literature, miR-129-5p was poorly expressed in patients with CHF [14]. Therein, we hypothesized that miR-129-5p may affect the CHF progression through the targeted regulation of Smurf1 expression. Dual-luciferase reporter gene experiments verified that compared with the mimic-NC group, the luciferase signal of WT-Smurf1 in the miR-129-5p mimic-transfected group was significantly decreased (*P* < 0.05). There was no significant difference in the luciferase activity in MUT-Smurf1 3ʹUTR (*P* > 0.05), indicating that miR-129-5p may specifically bind to Smurf1 (Figure 3(b)). Then, qRT-PCR showed notably decreased miR-129-5p in the myocardial tissue of the CHF group (*P* < 0.05) (Figure 3(c)). Smurf1 expression pattern in the miR-129-5p mimic group was markedly diminished compared with the mimic-NC group. Compared with the inhibitor-NC group, the Smurf1 expression in the miR-129-5p inhibitor group was upregulated (*P* < 0.05) (Figure 3(d,e)). Overall, these findings illustrated that miR-129-5p Smurf1 miR-17-5p expression.

### 3.4. miR-129-5p can significantly reverse the damaging effect of Smurf1 on cardiac function in CHF rats

**Figure 4.**
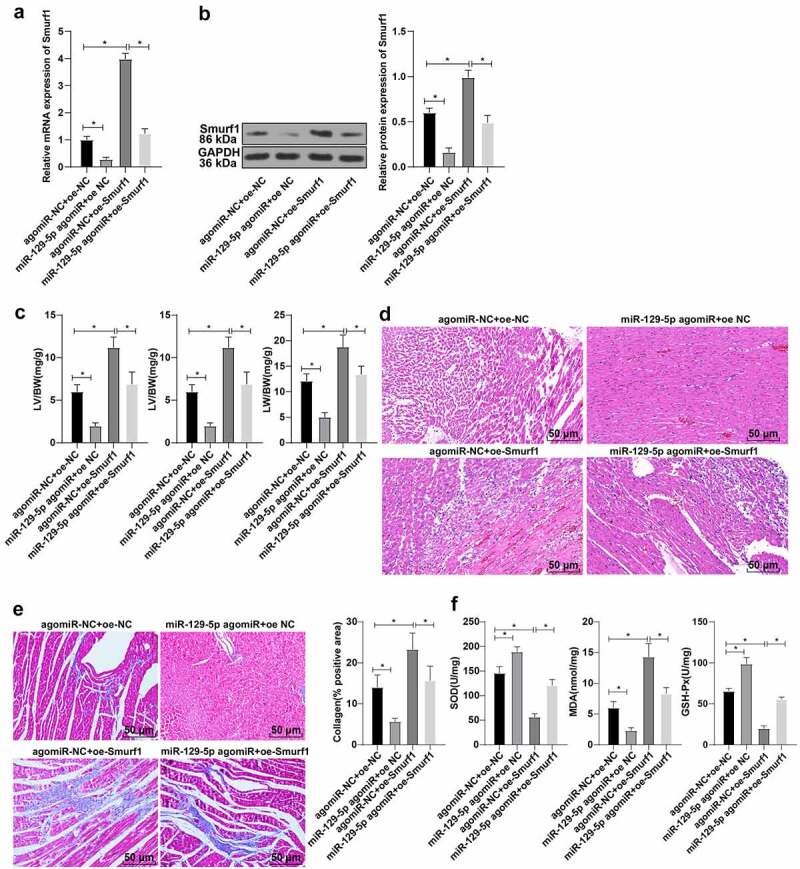
miR-129-5p affected cardiac function in CHF rats through Smurf1. A-B: The levels of Smurf1 in myocardial tissue of rats detected by qRT-PCR and WB; C: The ratios of heart weight/body weight, left ventricle weight/body weight, and lung weight/body weight were measured; D: Histopathological changes of the myocardium observed by HE staining; E: Masson staining observed the degree of myocardial tissue fibrosis; F: The levels of SOD, GSH-Px and MDA detected by ELISA; N = 10; Values in the figure were measurement data and displayed as mean ± standard deviation. Unpaired *t* test was used for pairwise comparisons; one-way ANOVA was used for comparisons among multiple groups, followed by Tukey’s test. * *P* < 0.05.

To further explore whether miR-129-5p affected the cardiac function in CHF rats through Smurf1, the rats were transfected and allocated into the following groups after modeling: agomiR-NC + oe-NC group, agomiR-NC + oe-Smurf1 group, miR-129-5p agomiR + oe-NC group, and miR-129-5p agomiR + oe-Smurf1 group. Compared with the agomiR-NC + oe-NC group, the Smurf1 expression levels were decreased in the miR-129-5p agomiR + oe-NC group, and Smurf1 was increased in the agomiR-NC + oe-Smurf1 group, while reduced in the miR-129-5p agomiR + oe-Smurf1 group relative to the agomiR-NC + oe-Smurf1 group (Figure 4(a,b)). Relative to the agomiR-NC + oe-NC group, the heart weight, left ventricular weight, and lung weight of rats in the miR-129-5p agomiR + oe-NC group were lowered, while the heart weight, left ventricular weight, and lung weight of rats were increased in the agomiR-NC + oe-Smurf1 group. Compared with the agomiR-NC + oe-Smurf1 group, the heart weight, left ventricular weight, and lung weight of miR-129-5p agomiR + oe-Smurf1 group were decreased (*P* < 0.05) (Figure 4(c)). HE staining revealed that the cross-sectional area of cardiomyocytes was shrunk and the myocardial structure was improved after simultaneous overexpression of miR-129-5p and Smurf1 (Figure 4(d)). Masson staining elicited that the fibrosis degree of myocardial tissue of rats in the miR-129-5p agomiR + oe-NC group was decreased compared with that in the agomiR-NC + oe-NC group, while the proportion of fibrosis area (blue area) to myocardial surface area (red area) and the fibrosis degree of myocardial tissue in the agomiR-NC + oe-SmurF1 group was increased; compared with the agomiR-NC + oe-Smurf1 group, the proportion of fibrosis region (blue area) to myocardial surface area (red area) and the degree of myocardial tissue fibrosis in the miR-129-5p agomiR + oe-Smurf1 group was significantly diminished (Figure 4(e)). Oxidative stress detection showed that relative to the agomiR-NC + oe-NC group, the levels of SOD and GSH-Px in the miR-129-5p agomiR + oe-NC group were enhanced and the MDA level was diminished, while the SOD and GSH-Px were decreased in the agomiR-NC + oe-Smurf1 group, and MDA was increased significantly; compared with the agomiR-NC + oe-Smurf1 group, SOD and GSH-Px in miR-129-5p agomiR + oe-Smurf1 group were increased, and MDA was decreased (*P* < 0.05) (figure 4(f)). Altogether, the aforementioned findings indicated that miR-129-5p can annul the damage of Smurf1 on cardiac functions in rats with CHF.

### 3.5. E3 ubiquitin ligase Smurf1 inhibited PTEN expression through ubiquitination degradation

**Figure 5.**
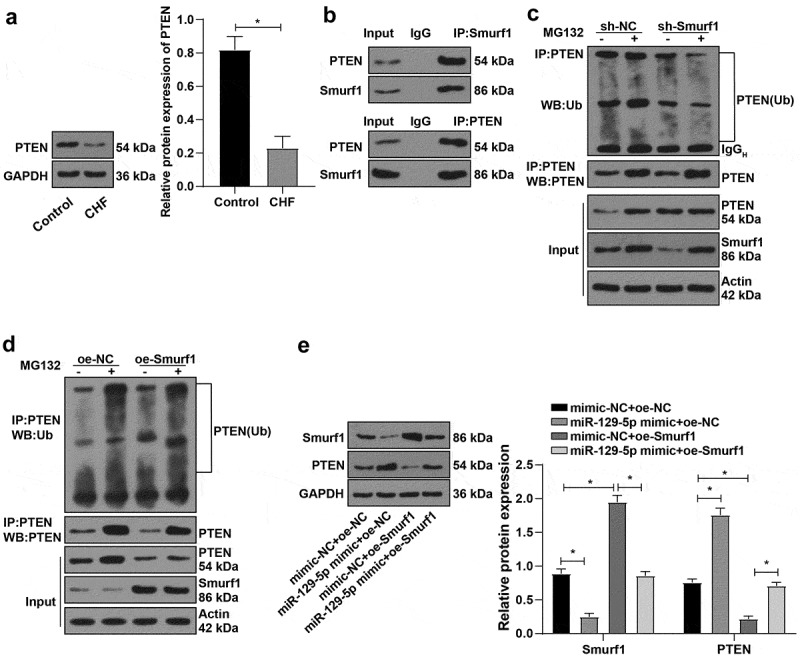
E3 ubiquitin ligase Smurf1 affects PTEN expression through ubiquitination. A: WB detected the PTEN protein level in myocardial tissues of CHF rats; B: IP experiment found that Smurf1 could be combined with PTEN; C: IP test was used to detect the changes of PTEN ubiquitination after Smurf1 silencing; D: The changes of PTEN ubiquitination after Smurf1 overexpression were detected by IP; E: Expressions of Smurf1 and PTEN in cells detected by WB; N = 10; Values in the figure were measurement data and displayed as mean ± standard deviation. Unpaired *t* test was used for pairwise comparisons; one-way ANOVA was used for comparisons among multiple groups, followed by Tukey’s test. * *P* < 0.05.

It has been reported that overexpression of Smurf1 promotes PTEN ubiquitination and degradation [28], and the expression of PTEN is down-regulated in the rat model of hypertrophic CHF [29]. Our experiment also confirmed the significantly reduced PTEN in the myocardial tissue of CHF rats (Figure 5(a)). Besides, IP experiments verified that Smurf1 could interact with PTEN in cardiomyocytes (Figure 5(b)). Meanwhile, the IP experiment revealed that PTEN ubiquitination was lowered and PTEN expression was enhanced after Smurf1 silencing (Figure 5(c)). However, after overexpression of Smurf1, PTEN ubiquitination was elevated, and PTEN was diminished; compared with the group without proteasome inhibitor MG132, the ubiquitination level of PTEN was decreased and the expression of PTEN was increased after adding MG132. (Figure 5(d)). Additionally, in the case of the overexpression of miR-129-5p and SmurF1 together, WB showed that Smurf1 was decreased and PTEN was significantly increased after the single overexpression of miR-129-5p. After the single overexpression of Smurf1, Smurf1 was increased and PTEN was reduced, and overexpression of miR-129-5p could reverse the effect of Smurf1 overexpression (Figure 5(e)). Collectively, Smurf1 inhibited PTEN expression by promoting PTEN ubiquitination, while miR-129-5p promoted PTEN expression by downregulating Smurf1.

### 3.6. Overexpression of PTEN can reverse the cardiac dysfunction in rats with CHF induced by Smurf1

To further validate whether PTEN as a downstream of Smurf1 to influence CHF, we divided the CHF rats into the oe-NC group, oe-Smurf1 + oe-NC group, oe-NC + oe-PTEN group, and oe-Smurf1 + OE-PTEN group.

**Figure 6.**
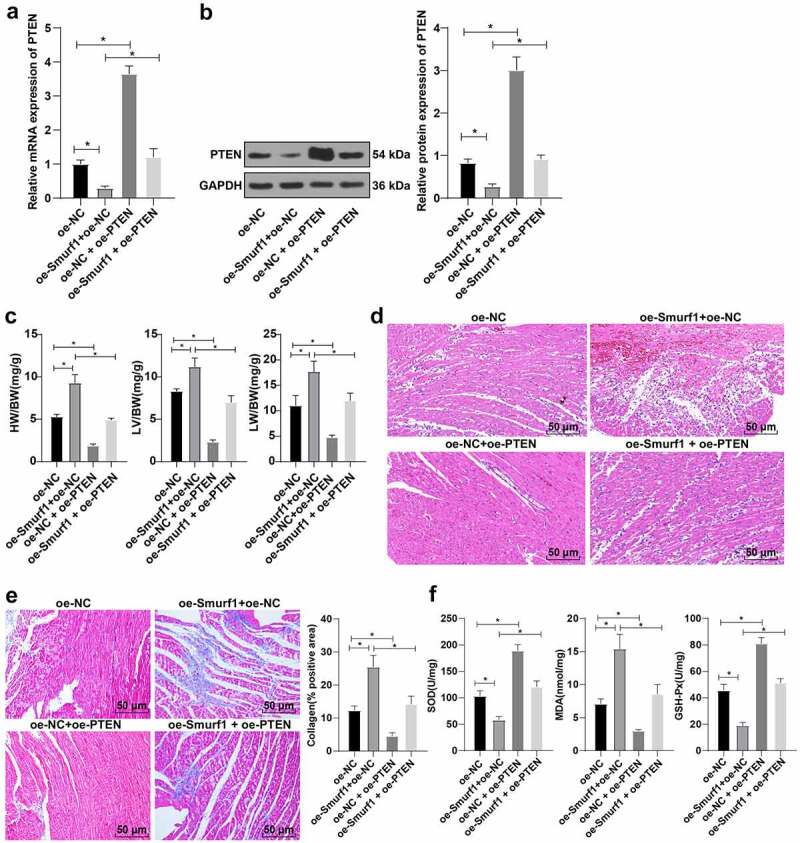
Smurf1 affects cardiac function in CHF rats through PTEN. AB: qRT-PCR and WB were used to detect the protein level of PTEN in myocardial tissues of CHF rats; C: The ratios of heart weight/body weight, left ventricle weight/body weight, and lung weight/body weight were measured; D: Histopathological changes of the myocardium observed by HE staining; E: Masson staining observed the degree of myocardial tissue fibrosis; F: The levels of SOD, GSH-Px and MDA detected by ELISA; N = 10; Values in the figure were measurement data and displayed as mean ± standard deviation. Unpaired *t* test was used for pairwise comparisons; one-way ANOVA was used for comparisons among multiple groups, followed by Tukey’s test. * *P* < 0.05.

RT-qPCR and WB showed that PTEN expression was lowered in the oe-Smurf1 + oe-NC group and up-regulated in the oe-NC + oe-PTEN group compared with the oe-NC group; Relative to the oe-Smurf1 + oe-NC group, the PTEN was raised in the oe-Smurf1 + OE-PTEN group (Figure 6(a,b)). Compared with the oe-NC group, the heart weight, left ventricular weight, and lung weight of rats in the oe-Smurf1 + oe-NC group was decreased, while the results were reversed in the oe-NC + oe-PTEN group. Compared with the oe-Smurf1 + oe-NC group, the heart weight, left ventricular weight, and lung weight were decreased in the oe-Smurf1 + oe-PTEN group (*P* < 0.05) (Figure 6(c)). HE staining showed that compared with the oe-NC group, the myocardial loss in the oe-Smurf1+ oe-NC group was aggravated, and the myocardial loss in the oe-NC + oe-PTEN group was further repaired; Relative to the oe-Smurf1 + oe-NC group, the cross-sectional area of cardiomyocytes in the oe-Smurf1 + oe-PTEN group was reduced with improved myocardial structure (Figure 6(d)). Masson staining showed that compared with the oe-NC group, myocardial fibrosis was aggravated in the oe-Smurf1 + oe-NC group, and the proportion of the myocardial tissue fibrosis area (blue area) to the myocardial surface area (red area) was reduced and the degree of myocardial fibrosis was decreased in the oe-NC + oe-PTEN group; The degree of myocardial tissue fibrosis in the oe-Smurf1 + oe-PTEN group was significantly decreased compared with the oe-Smurf1 + oe-NC group (Figure 6(e)). Compared with the oe-NC group, the levels of SOD and GSH-Px in the oe-Smurf1 + oe-NC group were decreased and the level of MDA was increased, while the levels of SOD and GSH-Px were elevated and the level of MDA was diminished in the oe-NC + oe-PTEN group. Compared with the oe-Smurf1 + oe-NC group, SOD and GSH-Px in the oe-Smurf1 + oe-PTEN group were elevated, while MDA was lowered (*P* < 0.05) (figure 6(f)). Overall, overexpression of PTEN reversed the cardiac dysfunction induced by Smurf1 in CHF rats.

## 4. Discussion

CHF, now a well-known public health concern, is the chief cause of death worldwide [5,14]. miRNAs play an essential role in cardiovascular disease, including heart failure [15]. Smurf1 is implicated in cardiovascular diseases through substrate ubiquitination [6,7]. Expanding our knowledge of the same, findings obtained in our study elucidated that miR-129-5p could target the ubiquitin ligase Smurf1 and promote the expression of PTEN, thus improving cardiac function in CHF rats.

Previous research has identified the involvement of Smurf1 in heart failure [7]. But its direct mechanism remains unclear. Firstly, the CHF rat model was established and we found the expression pattern of Smurf1 in CHF rats was increased. Smurf1 is highly expressed in outflow tract cushion mesenchyme involved in mammalian heart development, as a negative regulator for cardiomyogenesis and a positive regulator for cardiac fibroblast differentiation [30]. To further identify the role of Smurf1 in CHF, the rats were treated with sh-Smurf1. Upon Smurf1 knockdown, the heart weight, left ventricular weight, and lung weight of the rats were reduced, and the cross-sectional area of cardiomyocytes was reduced, and the degree of fibrosis of myocardial tissue was significantly decreased. The Smurf1/Smad6 complex antagonizes the myocardial fibrosis signal enhanced by TGF-β1/rock [31]. ROS is the main substance that induces oxidative stress in the body, and its overproduction can impair cardiac function and increase the susceptibility to arrhythmia, cause cardiomyocyte necrosis and apoptosis through direct toxicity, and trigger an inflammatory response [32]. Oxidative stress is vital in the pathogenesis of heart failure [33]. There is much evidence to highlight that heart failure could be relieved via inhibiting oxidative stress [34]. Interestingly, subsequent experimentation in our study confirmed revealed that the levels of SOD and GSH-Px were raised and MDA was lowered after Smurf1 knockdown. Reducing the level of Smurf1 in rats can inhibit the production of reactive oxygen species [35]. To the best of our knowledge, our study was the first-of-its-kind to suggest that interference with Smurf1 can inhibit oxidative stress and improve cardiac function in CHF rats.

Subsequently, we focused our efforts on elaborating the upstream mechanism of Smurf1. The Starbase website predicted the targeted binding sites between miR-129-5p and Smurf1. miR-129-5p is decreased in CHF patients [14]. Dual-luciferase assay verified that miR-129-5p might bind to Smurf1. Additionally, miR-129-5p was reduced in the myocardium of CHF rats. We concluded that miR-129-5p may affect the CHF progression through the targeted regulation of Smurf1 expression. To identify whether miR-129-5p affects cardiac functions in CHF rats through Smurf1, the rats were treated with miR-129-5p overexpression and/or Smurf1 overexpression. Our results elicited that upon both miR-129-5p and Smurf1 overexpression, the cardiac function of rats was damaged, the fibrosis in the myocardial tissue was relieved, the levels of SOD and GSH-Px were increased, while MDA was significantly reduced. Consistently, a prior study supports that miR-129-5p can improve the cardiac function of CHF rats and inhibit the apoptosis of cardiomyocytes [14]. miR-129-5p effectively inhibits Ang II–induced cardiac hypertrophy and oxidative stress [16]. Briefly, the aforementioned findings and evidence indicated that miR-129-5p can annul Smurf1-induced cardiac dysfunction in CHF rats.

According to a previous report, overexpression of Smurf1 promotes the ubiquitination degradation of PTEN [28]. Up-regulation of miR-132 can improve cardiac dysfunction and alleviate myocardial fibrosis in heart failure by inhibiting the expression of PTEN [36]. Our results verified that PTEN was decreased in the myocardium and Smurf1 could bind to PTEN in cardiomyocytes. Besides, PTEN ubiquitination was decreased and PTEN expression was increased after Smurf1 silencing. Overexpression of Smurf1 showed the opposite results. Meanwhile, Smurf1 was decreased and PTEN was enhanced after the single overexpression of miR-129-5p, indicating the overexpression of miR-129-5p could reverse the effect of overexpression of Smurf1. More notably, existing evidence suggests that Smurf1 promotes tumor progression through PTEN [8]. The timosaponin AIII contributes to the increase of miR-129-5-p, thus promoting PTEN expression [37]. Altogether, Smurf1 inhibited the expression of PTEN by promoting PTEN ubiquitination, while miR-129-5p promoted the PTEN by repressing Smurf1.

Recently, it has been proposed that PTEN can be regulated by post-translational modifications (such as ubiquitination), and the ectopic expression of Smurf1 significantly increases the ubiquitinated PTEN protein level [28]. To explore whether PTEN as a downstream of Smurf1 affects CHF, we overexpressed Smurf1 and PTEN in CHF rats. The subsequent experimental results showed that when Smurf1 and PTEN were overexpressed simultaneously, PTEN was increased in rats, ventricular hypertrophy, pulmonary edema, and the myocardial structure were improved, fibrosis of myocardial tissue in rats was decreased, and oxidative stress was significantly relieved. Further in line with our findings, a prior study indicated that adverse cardiac systolic and diastolic function, fibrosis, and oxidative stress can be significantly reduced by inhibiting PTEN degradation and downstream mediators [38]. These results indicated that overexpression of PTEN can reverse the cardiac dysfunction induced by Smurf1 in rats with CHF.

Collectively, our findings indicated that miR-129-5p can improve cardiac function of CHF rats by targeting E3 ubiquitin ligase Smurf1 and promoting the expression of PTEN. However, due to the limited time and funds, the expression patterns and mechanisms of miR-129-5p, Smurf1, and PTEN in the cell models and the clinical setting are not investigated. Therefore, further studies should be conducted on the cell level to find out multiple mechanisms of miR-129-5 on CHF, so as to offer novel insights into CHF management.

## 5. Conclusion

In summary, this study suggested that the protective effect of miR-129-5p overexpression on CHF is mainly reflected in the improvement of cardiac function, anti-myocardial fibrosis, and inhibition of oxidative stress in rats. In addition, the key mechanism of miR-129-5p acting on CHF was revealed, that is, by targeting Smurf1, miR-129-5p prevented the ubiquitination of PTEN and promoted the expression of PTEN, thus improving the cardiac function of CHF. Taken together, miR-129-5p has great potential in the treatment of CHF and is expected to become a new target for the treatment of CHF.

## Data Availability

All the data generated or analyzed during this study are included in this published article.
